# Brain Alterations and Clinical Symptoms of Dementia in Diabetes: Aβ/Tau-Dependent and Independent Mechanisms

**DOI:** 10.3389/fendo.2014.00143

**Published:** 2014-09-05

**Authors:** Naoyuki Sato, Ryuichi Morishita

**Affiliations:** ^1^Department of Clinical Gene Therapy, Graduate School of Medicine, Osaka University, Osaka, Japan; ^2^Department of Geriatric Medicine, Graduate School of Medicine, Osaka University, Osaka, Japan

**Keywords:** dementia, diabetes mellitus, Alzheimer disease, abeta, tauopathies

## Abstract

Emerging evidence suggests that diabetes affects cognitive function and increases the incidence of dementia. However, the mechanisms by which diabetes modifies cognitive function still remains unclear. Morphologically, diabetes is associated with neuronal loss in the frontal and temporal lobes including the hippocampus, and aberrant functional connectivity of the posterior cingulate cortex and medial frontal/temporal gyrus. Clinically, diabetic patients show decreased executive function, information processing, planning, visuospatial construction, and visual memory. Therefore, in comparison with the characteristics of AD brain structure and cognition, diabetes seems to affect cognitive function through not only simple AD pathological feature-dependent mechanisms but also independent mechanisms. As an Aβ/tau-independent mechanism, diabetes compromises cerebrovascular function, increases subcortical infarction, and might alter the blood–brain barrier. Diabetes also affects glucose metabolism, insulin signaling, and mitochondrial function in the brain. Diabetes also modifies metabolism of Aβ and tau and causes Aβ/tau-dependent pathological changes. Moreover, there is evidence that suggests an interaction between Aβ/tau-dependent and independent mechanisms. Therefore, diabetes modifies cognitive function through Aβ/tau-dependent and independent mechanisms. Interaction between these two mechanisms forms a vicious cycle.

## Introduction

More than 30 million patients suffer from dementia (1), while 285 million battle diabetes in this aging society (2). Emerging evidence suggests that diabetes increases the incidence of dementia (3–8). Indeed, diabetes in mid-life is associated with mild cognitive impairment (MCI) (3), and impaired glycemia increases disease progression to dementia in patients with MCI (4). Moreover, numerous epidemiological studies have also demonstrated that patients with diabetes have a significantly higher risk of developing AD (5–8). While genetic and non-genetic risk factors contribute to sporadic AD (9), APOEε4 is the strongest genetic risk factor for sporadic AD and is believed to promote the development of senile plaques. However, the mechanisms by which diabetes modifies cognitive function still remain unclear (10). Here, we review recent advances in brain alterations and clinical symptoms in dementia associated with diabetes and its Aβ/tau-dependent and independent mechanisms.

## Brain Alterations in Dementia Associated with Diabetes

Diabetes causes functional and structural deficits in the brain (Table 1). Diabetes is associated with reduced volume of the hippocampus (3, 11), whole brain (3), gray (12), and white matter (11). Gray matter loss is distributed in the medial temporal, anterior cingulate, and medial frontal lobes (3, 11, 12), and white matter loss occurs in the frontal and temporal regions (11), whereas, in AD, gray matter loss is in temporal lobe, hippocampus, entorhinal, and parietal lobes (13–15), and white matter loss is in the temporal region (16). In addition to neuronal loss, diabetes also affects functional connectivity. Resting-state functional connectivity, measured by functional MRI, is used to access brain function. Disruption of resting-state functional connectivity in the default mode network, which is most active during rest, is recently believed to be a predictor of current and future cognitive dysfunction, especially in AD (17, 18). Diabetic patients develop aberrant functional connectivity of the posterior cingulate cortex with the medial temporal gyrus (19) and medial frontal gyrus (20), reflecting white matter abnormalities in diabetes. Diabetic patients also have decreased spontaneous brain activity in the occipital lobe and postcentral gyrus (21). Moreover, functional MRI during task performance demonstrates that diabetic patients show reduced activation of the dorsolateral prefrontal cortex during encoding (22). These studies suggest that diabetes is associated with neuronal loss in the frontal and temporal lobes including the hippocampus, and aberrant functional connectivity between the posterior cingulate cortex and medial frontal/temporal gyrus.

**Table 1:** **Comparison of brain structural and functional alteration in diabetes and Alzheimer disease**.

		Diabetes	Alzheimer disease
Structural change	Gray matter loss	Frontal, temporal lobes, hippocampus (3, 11, 12), anterior cingulate cortex (11)	Temporal lobe, hippocampus, entorhinal, and parietal lobes (13–15)
	
	White matter loss	Frontal and temporal regions (11)	Temporal region (16)

Functional change	Aberrant functional connectivity	Between posterior cingulate cortex and medial frontal (20)/temporal (19) gyrus	Between posterior cingulate cortex and hippocampus in medial temporal lobe (17, 18)
	
	Decreased spontaneous brain activity	Occipital lobe and postcentral gyrus (21)	Posterior cingulate cortex, medial temporal lobe (23)
	
	Task-induced brain activity	Reduced activation of dorsolateral prefrontal cortex during encoding and deactivation of default mode network during recognition (22)	Reduced activation in hippocampal formation but increased activation in medial parietal and posterior cingulate regions during encoding (24)

## Clinical Symptoms in Dementia Associated with Diabetes

**Figure 1:**
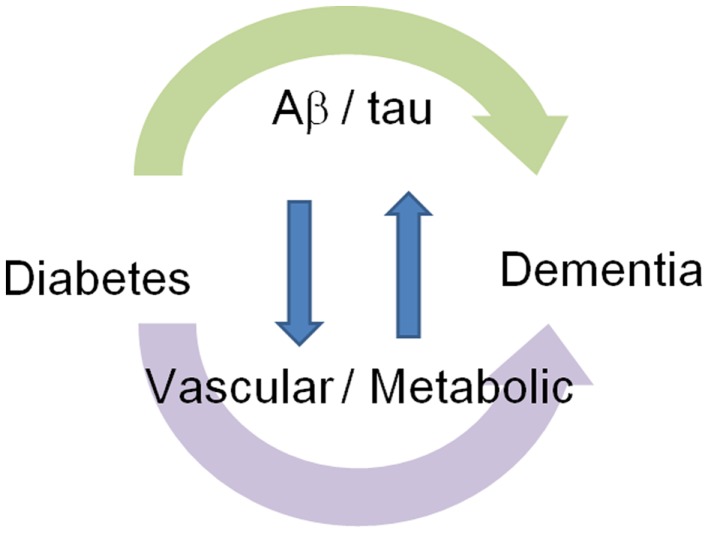
**Association of dementia with diabetes: Aβ/tau-dependent and independent mechanisms**. Diabetes modifies cognitive function through Aβ/tau-dependent and independent mechanisms. Interaction between these two mechanisms forms a vicious cycle.

Diabetic patients show impaired cognitive function, increased behavioral symptoms, and decreased activity of daily living (Table 2). Diabetic patients show impairment of memory (25), attention (26, 27), executive function (28, 29), information processing (28, 29), planning (11), visuospatial construction (11), and visual memory (11, 21). Diabetic patients are reported to have impaired memory retrieval rather than encoding (25). Patients with higher HbA1c have increased behavioral and psychological symptoms (30) such as apathy, overeating, and excessive daytime sleeping, and also have impaired activities of daily living (30). Therefore, the knowledge about brain alterations and clinical symptoms suggests that diabetes affects cognitive function through not only simply AD pathological feature-dependent mechanisms but also independent mechanisms (Figure 1).

**Table 2:** **Comparison of cognitive and behavioral alterations in diabetes and Alzheimer disease**.

		Diabetes	Alzheimer disease
Cognitive function	Memory	Decreased (25)	Decreased (31, 32)
	
	Attention	Decreased (26, 27)	Decreased (31, 32)
	
	Executive function	Decreased (28, 29)	Decreased (32)
	
	Information processing	Decreased (28, 29)	Decreased (33), but less in initial phase
	
	Planning	Decreased (11)	Decreased (34)
	
	Visuospatial construction	Decreased (11)	Decreased (35, 36)
	
	Visual memory	Decreased (11, 21)	Decreased (37)

Behavioral psychological symptom	Apathy	Increased (30)	Increased (38)
	
	Overeating	Increased (30)	Increased (39), but less in initial phase
	
	Excessive daytime sleeping	Increased (30)	Increased (40)

Activity of daily living	–	Decreased (30)	Decreased (41)

## Alzheimer Pathological Feature-Independent Mechanisms

### Vascular mechanism

Cerebrovascular damage is related to cognitive function (42–45) and brain atrophy (46). Diabetes is associated with vascular reactivity impairment (47, 48), microangiopathy (49), and cerebrovascular lesions (50), including subcortical infarcts (3). Indeed, microvascular network alteration in the retina, which is believed to be a predictor of vascular changes inside the brain, is associated with increased risk of cognitive dysfunction (51) and AD (52) in diabetes. Vascular permeability is reported to be increased in diabetic retinopathy (53), suggesting that diabetes disrupts the blood–brain barrier (BBB) inside the brain. These data suggest that diabetes compromises cerebrovascular function, increases subcortical infarction, and might cause BBB dysfunction.

### Metabolic mechanism

#### Hyperglycemia and hypoglycemia – brain glucose metabolism

Hyperglycemia and hypoglycemia have impacts on cognitive function and activity of daily life. Interestingly, Holmes et al. demonstrated that attention and fine motor skills were slowed at altered glucose levels, assessed in diabetic patients during hypoglycemia and hyperglycemia induced by an artificial insulin/glucose infusion system (27). In the long term, the duration of diabetes is associated with impaired cognition in patients with higher HbA1c levels (54). Glucose metabolism declines with age in many brain regions (55), and glucose hypometabolism and brain atrophy are associated with concurrent cognitive dysfunction (56).

Hypoglycemia is associated with cognitive impairment in elderly diabetic patients (57). Because the brain uses mainly glucose as an energy source, hypoglycemia causes defects of neuronal function, though lactate can also be used in such situations (58, 59). In addition to dysfunction of individual cells, failure of neuronal networking also contributes to cognitive impairment in a hypoglycemic state (60). In the long term, repeated episodes of severe hypoglycemia are reported to also be a risk for the development of dementia (61). Hyperglycemia affects cognitive function, and is associated with brain hypometabolism (62), impaired deactivation of the default mode network (22), poorer memory, and reduced hippocampal microstructures (63). Behavioral and psychological symptoms, including apathy, overeating, and excessive daytime sleeping, appear to be increased in patients with HbA1c ≥7.0% (30). In an animal model, hyperglycemia induced by a high-fat diet causes chronic energy imbalance with resulting loss of neurons and reduces olfactory learning (64). These studies suggest that hyperglycemia has an impact on cognition and behavior through glucose-energy imbalance. Although the memory in diabetes (MIND) sub-study of action to control cardiovascular risk in diabetes (ACCORD) suggests that intensive glycemic control has no effect on cognitive function (65), the incidence of hypoglycemia should be considered in interpreting the data (66).

#### Hyperinsulinemia – brain insulin signaling

Insulin signaling is believed to be decreased in the diabetic brain. Insulin receptors are expressed in the cortex and hippocampus (67–69), and peripheral insulin accesses the brain by crossing the BBB (70). Insulin is also produced in the CNS. Indeed, single-cell PCR reveals that insulin is strongly expressed in GABAergic neurogliaform cells in the cerebral cortex (71). Similarly, insulin-like growth factor-1 (IGF-1), IGF-2, and their receptors exist in the CNS (72–75). When insulin binds to the insulin receptor, IRS-1 and -2 (insulin receptor substrate) undergo tyrosine phosphorylation and bind phosphatidylinositol 3-kinase (PI3K) (76), which activates AKT and glycogen-synthase kinase-3β (GSK3β) (77–79). Importantly, insulin receptor knockout mice show no obvious alteration in the brain (80), suggesting compensation of IGF receptor signaling for insulin signaling. Liu also reported that the levels and activities of several components of the insulin–PI3K–AKT signaling pathway were decreased in patients with diabetes (81). Taken together, these findings indicate that insulin/IGF signaling is impaired in the diabetic brain, and this signaling might have an impact on aging-related brain dysfunction (82). Interestingly, aerobic exercise increases some proteins related to the insulin/IGF-1 pathway in the hippocampus and improves spatial memory in diabetic rats (83).

#### Brain mitochondrial metabolism

Diabetes deregulates mitochondrial function in mouse (84) and rat (85) neurons. Recent work reveals that hyperglycemia mediates a phenotypic change in mitochondrial biology through alteration of AMP-activated protein kinase (AMPK), a key cellular energy sensor that regulates the activity of a number of metabolic enzymes (86). It is known that an anti-diabetic drug, metformin, activates AMPK kinase. These findings might explain the recent clinical observation that use of metformin is associated with increased risk of cognitive impairment in patients with diabetes (87), though this is still controversial (88). Diabetes is also reported to impair neurite outgrowth through JAK/STAT3 modulation of mitochondrial bioenergetics in neurons (89). Thus, mitochondria might be a new therapeutic target not only for diabetic neuropathy (90) but also for dementia associated with diabetes.

## Alzheimer Pathological Feature-Dependent Mechanisms

### Aβ-dependent mechanism

While AD consists of both familial and sporadic forms, familial AD is caused by mutations in the amyloid precursor protein (91) and presenilin (92). Both mutations cause overproduction of Aβ, particularly its longer form, Aβ42, which is more prone to aggregate *in vitro* (93) and deposits first in the brain (94) to form senile plaques. Insulin resistance in mid-life is associated with the development of senile plaques (8), though retrospective studies suggest that the magnitude of senile plaques and another hallmark, neurofibrillary tangles, is comparable between AD with and without diabetes (95). Several groups report that a high-fat diet causes Aβ accumulation in the brain of wild type rabbits (96) and APP Tg mice (97, 98). Accumulation of autophagosomes to enhance amyloidogenic APP processing (99) or up-regulation of BACE1 (100) have thus far been proposed as the mechanisms of the increase in Aβ by diabetes. APP^+^-*ob*/*ob* mice, produced by crossing of diabetic and obese *ob*/*ob* mice, manifest no change in total brain Aβ level, but increase Aβ deposition in the cerebral vasculature (101). APP^+^-*ob*/*ob* mice show up-regulation of RAGE, the receptor for AGE (102), in the vasculature. Because RAGE mediates amplification of inflammatory responses (103), inflammatory cytokines are upregulated around the cerebrovasculature in APP^+^-*ob*/*ob* (101). Soluble Aβ itself is believed to reduce endothelial function *in vitro* (27) and vascular reactivity in mice (104) and humans (105, 106). Interestingly, crossing obese and diabetic *db/db* mice with APP/PS1 knock-in mice leads to severe cerebrovascular pathological features, including aneurysms and small strokes, though no further Aβ deposition in the vasculature, indicating an interaction between soluble Aβ and a diabetic vascular factor (107).

Glucose-energy metabolism is altered in AD (108) and various AD models (109–111). Amyloid burden is accompanied by glucometabolic increases in people at risk for AD (108). Brain 18FDG uptake is a sensitive biomarker for early detection of abnormal metabolism in the 5XFAD mouse (109), indicating that glucose metabolism is decreased in the AD model brain. A glucose transporter, Glut-1, is reduced in the brain capillaries of 18-month-old 3xTg-AD mice (110), suggesting that glucose uptake into the brain might be altered. Another AD model, APP/PS1 mice, also shows alterations in energy-sensor AMPK (111). AMPK could mediate the toxic effects of Aβ through tau phosphorylation (112).

Insulin signaling is also altered by Aβ. In the AD brain, the levels of insulin and IGF (113) and the responses to insulin and IGF (114) are reduced. The levels and activities of the insulin signaling pathway are also decreased in AD (81, 115) and diabetic brains (81), as mentioned above. AD animal models show significant reductions in insulin receptor (111), IRS-1 (116), and IRS-2 (111) in the brain. Therefore, diabetes and Aβ could synergistically affect insulin signaling (101, 117, 118). This impaired insulin signaling could lead to an increase of tau phosphorylation.

### Tau-dependent mechanism

Diabetes could promote tau phosphorylation, and then formation of neurofibrillary tangles, which is one of the major pathological features of AD. Normal tau promotes the assembly and stabilization of microtubules, but abnormally hyperphosphorylated tau sequesters normal tau and disrupts microtubules (119, 120). Several neuropathological studies suggest that the magnitude of neurofibrillary tangles in the brain at autopsy is not different between AD with and without diabetes (95). However, animal studies show that tau phosphorylation is increased in diabetes (121–125). Tau phosphorylation is increased in the cortex and hippocampus in *db/db* mice (121) and streptozotocin-induced diabetic mice (122–124). Moreover, streptozotocin exacerbates neurofibrillary tangles in a transgenic mouse model over-expressing the P301L mutant human tau (125). Recently, Takalo et al. report that a high-fat diet induces the expression of four repeat tau mRNA and protein in the temporal cortex (126). Importantly, tau phosphorylation sites in AD are shown to be increased in the diabetic human brain (127). These data suggest that diabetes promotes tau phosphorylation, splicing, and the formation of neurofibrillary tangles.

Impaired insulin signaling in the brain could cause tau phosphorylation (128) and insulin signaling is mainly mediated through the PI3K–AKT–GSK3β pathway (77–79). Because GSK3β phosphorylates tau, insulin inhibits tau phosphorylation through negative regulation of GSK3β (129). Therefore, loss of insulin (130), insulin receptor (80), or IRS-2 (131–133) increases tau phosphorylation. While protein phosphorylation is also regulated by kinases and phosphatases, tau is reported to be dephosphorylated by protein phosphatase 2A (PP2A) (134). As disruption of IRS-2 downregulates PP2A (134), impaired insulin signaling might cause tau phosphorylation by influencing not only kinases but also phosphatases. Importantly, depletion of endogenous tau mitigates behavioral impairment and synaptic deficits induced in diabetic mice (135). Taken together, these findings indicate that diabetes could promote tau phosphorylation via impaired insulin signaling in the brain and then, contribute to cognitive impairment.

## Conclusion

Morphologically, diabetes is associated with neuronal loss in the frontal and temporal lobes including the hippocampus, and aberrant functional connectivity of the posterior cingulate cortex and medial frontal/temporal gyrus, and decreased spontaneous brain activity in the occipital lobe and postcentral gyrus. As clinical symptoms, diabetic patients show decreased executive function, information processing, planning, visuospatial construction, and visual memory. Therefore, in comparison with the characteristics in AD brain structure and cognition, diabetes seems to affect cognitive function through not only simply AD pathological feature-dependent but also independent mechanisms. As Aβ/tau-independent mechanisms, diabetes compromises cerebrovascular function, increases subcortical infarction, and might alter BBB. Diabetes also compromises glucose metabolism, insulin signaling, and mitochondrial function. Diabetes also modifies metabolism of Aβ and tau and causes Aβ/tau-dependent pathological changes. Interestingly, in cognitively normal diabetic subjects, higher mean HbA1c levels are associated with lower cognitive performance in ApoEε4 carriers (136), indicating an interaction between Aβ/tau-dependent and independent mechanisms. In conclusion, diabetes modifies cognitive function through Aβ/tau-dependent and independent mechanisms. Interaction between these two mechanisms forms a vicious cycle.

## Conflict of Interest Statement

The authors declare that the research was conducted in the absence of any commercial or financial relationships that could be construed as a potential conflict of interest.

## Abbreviations

AMPK, AMP-activated protein kinase; APP, amyloid precursor protein; BBB, blood–brain barrier; IGF-1, insulin-like growth factor-1; IRS-2, insulin receptor substrate-2; MCI, mild cognitive impairment; RAGE, receptor for advanced glycation endproducts.
